# Folic Acid-Coated Nanochitosan Ameliorated the Growth Performance, Hematological Parameters, Antioxidant Status, and Immune Responses of Rainbow Trout (*Oncorhynchus mykiss*)

**DOI:** 10.3389/fvets.2021.647722

**Published:** 2021-06-15

**Authors:** Sahar Farahnak Roudsari, Houman Rajabi Islami, Seyed Abdolmajid Mousavi, Mehdi Shamsaie Mehrgan

**Affiliations:** ^1^Department of Fisheries, Science and Research Branch, Islamic Azad University, Tehran, Iran; ^2^Department of Animal Science, Varamin-Pishva Branch, Islamic Azad University, Varamin, Iran

**Keywords:** chitosan, nanoparticle, folic acid, rainbow trout, innate immunity

## Abstract

In recent years, chitosan has gained considerable attention due to its favorable properties such as excellent biocompatibility and biodegradability for which it can be used as a health supplement for delivering bioactive compounds in the food industry and nutrition. In the present study, the effect of nanochitosans coated with folic acid (FA) was considered on the growth performance, hematological parameters, antioxidant status, and serum immune responses of rainbow trout (*Oncorhynchus mykiss*) fingerlings. Graded levels of FA-coated nanochitosan (0, 0.1, 0.25, 0.5, and 1 mg kg^−1^ diet) were added to the basal diet, and each experimental diet was fed to three groups of fish with an approximate weight of 31 g for 8 weeks. The experimental study demonstrated that dietary FA-coated nanochitosan significantly (*P* < 0.05) improved the weight gain ration (WGR), specific growth rate (SGR), and feed conversion ratio (FCR) of fish at the end of the feeding trial. There were also linearly increasing trends in red blood cells (RBCs), white blood cells (WBCs), hemoglobin (Hb), and hematocrit (Hct) of fish fed with increasing dietary chitosan/FA levels, whereas no significant difference was recorded in differential leukocyte count of fish among the treatments. In case of antioxidant responses, fish fed diet supplemented with 0.50 mg kg^−1^ FA-coated nanochitosan had the highest CAT and SOD activities, while the maximum activity of GPX was found in fish fed diet supplemented with 1.00 mg kg^−1^ FA-coated nanochitosan. Malondialdehyde activity also reached the lowest value in fish fed with 1.00 mg kg^−1^ FA-coated nanochitosan-supplemented diet (*P* < 0.05). Measured immune responses showed a linear augmentation in lysozyme activity (LA) with increasing dietary FA-coated nanochitosan, while linearly and quadratically increasing trends were recorded in immunoglobulin M (IgM) content as well as complement component C3 and C4 activities by increasing the supplementation of nanochitosan coated with FA (*P* < 0.05). Findings of the current study illustrated the positive effect of dietary FA-coated nanochitosan as a promising compound on improving the growth performance, feed utilization, antioxidant status, and immune responses of rainbow trout.

## Introduction

Folic acid (FA) is the completely oxidized form of vitamin B9 that plays essential roles in key metabolic pathways involved in purine and pyrimidine synthesis, amino acid metabolisms, methylation reactions, and formate generation (1, 2). It consists of an aromatic pteridine ring linked through a methylene bridge to para-aminobenzoic acid and glutamate to form FA (3), which acts in a variety of physiological processes such as cell multiplication, protein methylation, gene activity regulation, and red blood cell creation after conversion to active tetrahydrofolate (4, 5).

In spite of its higher bioavailability than that of naturally occurring folate (6), FA is relatively sensitive to environmental conditions (heat, oxidation, pH) and readily decomposes during the cooking process or normal storage temperature of farmed fish feed (7, 8). Deficiency of FA may cause reduced growth, poor feed efficiency, appetite loss, macrocytic normochromic, megaloblastic anemia, spleen infarction, and higher sensitivity to bacterial infections (9–11). Accordingly, the quantitative requirement of FA has been determined for different fish species such as rainbow trout, *Oncorhynchus mykiss* (12), Nile tilapia, *Oreochromis niloticus* (13), juvenile grouper, *Epinephelus malabaricus* (14), grass carp, *Ctenopharyngodon idella* (9), blunt snout bream, *Megalobrama amblycephala* (15), spotted snakehead, *Channa punctata* (10), and Siberian sturgeon, *Acipenser baerii* (16).

Among the cultural fish species, rainbow trout is one of the most important farmed fish natively distributed in the cold-water rivers and lakes of the North Pacific coast (17, 18). High meat quality, simple farming, disease resistance, and reliable reproduction under cultural conditions made this species the most widely introduced fish in the world (19). Based on the latest published statistics, about 814 thousand tons of rainbow trout are cultured by 80 countries such as Japan, Iran, United States, Chile, Norway, Denmark, Turkey, Italy, and Germany with the global production value of more than 3.6 million U.S. dollars (20).

Nanoparticles are assigned to solid microscopic particles or particulate dispersions which have the size range of 1–100 nm for at least one dimension (21). In the recent years, considerable attention has been paid to develop biodegradable nanoparticles as effective components for delivering many bioactive feed compounds such as antioxidants, natural pigments, fatty acids, antimicrobial agents, phenolic compounds, and vitamins (22, 23). Due to its ultrafine size, appropriate stability, very low toxicity, and exclusive functional groups for a wide range of entrapments, chitosan has gained a large amount of interest to be used as a delivery vehicle for bioactive molecules such as hydrophobic drugs, vitamins, proteins, nutrients, and phenolic compounds into the biological systems (24, 25). This natural biopolymer is a linear cationic polysaccharide composed of randomly distributed N-acetyl-D-glucosamine and N-acetyl-d-glucosamine monomers that linked together through β-,4-glycosidic bonds (26). Commercially, chitosan is produced by alkaline deacetylation of chitin, the second most abundant natural biopolymer that forms the exoskeletons of crustaceans (27). Accordingly, the present study was designed to assess the effects of nanochitosan coated with folic acid on growth performance, feed utilization, body composition, antioxidant parameters, and immune responses of rainbow trout.

## Materials and Methods

### Materials

Low molecular weight (50–190 kDa) chitosan with a deacetylation degree of 75–85% was purchased from Sigma-Aldrich, St-Louis, MO, USA. Sodium tripolyphosphate (TPP) was also obtained for Merck KGaA, Darmstadt, Germany. All reagents were commercially available and used without any modification.

### Nanoparticle Preparation and Physiochemical Evaluation

Chitosan nanoparticles containing FA were prepared according to Alishahi et al. (28) with some modification. A chitosan solution was made by dissolving chitosan in a 1% (w/v) acetic acid solution using a magnetic stirrer to prepare a transparent solution. After complete dissolution, the solution was diluted by adding deionized water to produce a chitosan solution with a final concentration of 0.01 (w/v), while the pH was reduced to 0.6 by dropwise addition of NaOH under constant stirring at a rate of 15,000 rpm. Simultaneously, TPP was dissolved in deionized water at a concentration of 0.1% (w/v). Under magnetic stirring for 15 min, the TPP solution was mixed with an equal volume of chitosan solution for spontaneous formation of chitosan–TPP nanoparticles via ionic gelatin mechanism induced by TPP. The nanoparticle suspension was then subjected to mid magnetic stirring for 60 min at room temperature and mixed with folic acid to prepare chitosan–TPP nanoparticles coated with FA via cross-linking between the positive-charged amino groups of chitosan–TPP and FA. The nanoparticles were then isolated by centrifuging the solution at 8,000 × g for 30 min at 4°C using a refrigerated centrifuge (3−30KS, Sigma Laborzentrifugen GmbH, Osterode am Harz, Germany) and lyophilizing the precipitate at −46°C using a freeze drier (Alpha 1-2 LDplus, Christ, Osterode am Harz, Germany).

For determination of encapsulation efficiency and loading capacity, the content of free FA in the supernatant was determined using a UV-vis spectrophotometer (Cary 100 UV-Vis, Agilent, Santa Clara, USA) at a wavelength of 303 nm with the supernatant of their corresponding blank nanoparticles without coated FA as basic correction (29). The EE (%) and LC (%) were then calculated by the following equations, respectively: EE = 100 × weight of initially added FA − weight of free FA in the supernatant / weight of initially added FA; LC = 100 × weight of initially added FA − weight of free FA in the supernatant / weight of sample.

Zeta potential and hydrodynamic size distribution of the nanoparticles were determined by dynamic light scattering technique using a Nano ZS Zetasizer (Malvern Instruments, Malvern, UK). The morphology of the nanoparticles was characterized using a field emission scanning electron microscope (TESCAN MIRA3, Tescan, Kohoutovice, Czech Republic). Fourier transform infrared (FTIR) spectra were recorded using a FTIR spectrophotometer (WQF-510, Beijing Rayleigh Analytical Instrument Corporation, Beijing, China) equipped with a deuterated triglycine sulfate (DTGS) detector and KBr beam splitter. Interferograms were accumulated over the spectral range between 4,000 and 400 cm^−1^ with a nominal resolution of 2 cm^−1^ and 100 scans.

### Diet Preparation

The formulation and proximate composition of the basal diet are shown in Table 1. Fish meal, soybean meal, and wheat gluten were used as protein sources, while soybean oil and fish oil were used as lipid sources. Five experimental diets were prepared from the purified ingredients by supplementing graded levels (0.0, 0.1, 0.25, 0.5, and 1 mg kg^−1^) of nanochitosans coated with FA at the expense of alpha cellulose. The diets were prepared as described by Jamalzad Falah et al. (16) and stored at −20°C until use.

**Table 1:** Ingredients and proximate compositions of the basal diet for rainbow trout, *Oncorhynchus mykiss*.

**Ingredients**	**g kg^**−1**^**
Kilka fish meal^a^	630.00
Soybean meal^b^	130.00
Wheat gluten^c^	20.00
Fish oil^d^	90.00
Soybean oil^b^	50.00
Alpha cellulose	30.00
Vitamin mixture (folic acid free)^e^	20.00
Mineral mixture^f^	30.00
**Proximate composition**	
Moisture	97.42
Crude protein	524.72
Crude lipid	174.28
Ash	123.91

a *Gill Powder Corporation, Anzali, Iran*.

b *Behpak Industrial Co., Behshahr, Iran*.

c *Golden flour Co., Tehran, Iran*.

d *Refined fish oil of Black Sea sprat, Clupeonella cultriventris, Khazar Oil Co. Ltd., Anzali, Iran*.

e *Composition per kg of mixture: vitamin A, 500,000 IU; vitamin D_3_, 400,000 IU; vitamin K_3_, 1,000 mg; vitamin E, 40,000 mg; vitamin B_1_, 5,000 mg; vitamin B_2_, 5,000 mg; pantothenic acid, 40,000 mg; niacin, 15,000 mg; vitamin B_6_, 3,000 mg; vitamin B_12_, 5,000 mg; antioxidant 8,000 mg; inositol, 25,000 mg; biotin, 240 mg; All ingredients were diluted with alpha cellulose to 1 kg*.

f *Composition per kg of mixture: zinc sulfate, 6,000 mg; iron sulfate, 4,000 mg; cobalt sulfate, 500 mg; sodium selenite, 500 mg; copper sulfate, 6,000 mg; potassium iodide, 2,000 mg; choline chloride, 120,000 mg; manganese sulfate, 2,600 mg; All ingredients were diluted with alpha cellulose to 1 kg*.

### Experimental Fish and Feeding Procedure

Healthy fingerling rainbow trout, *Oncorhynchus mykiss*, were obtained from a local farm in Fiurozkoh, Tehran, Iran, and transferred to an indoor culture system for adaptation to the experimental conditions before commencement of the feeding trial. During the 2-week acclimatization period, fish were fed with the basal diet to apparent satiation for depleting possible FA reserved in their body tissues. At the beginning of the experiment, fish of similar sizes (~31 g) were randomly stocked into fifteen 500-L polyvinyl tanks (filled with 400 L water) with 25 fish per tank. Each experimental diet was assigned to three tanks in a completely randomized design (each tank as a replicate group). The experimental tanks were supplied with continuously aerated freshwater in a flow-through system at a water change rate of 20% h^−1^. The fish were fed daily with experimental diets to apparent satiation four times (07:00, 11:00, 15:00, and 19:00 h) over 8 weeks. Any dead fish were daily removed and not replaced during the experiment. During the acclimation period and feeding trial, the water temperature was 16.5 ± 0.5°C, dissolved oxygen 7.9 ± 0.2 mg L^−1^, pH 6.97 ± 0.10, and negligible levels of free ammonia and nitrite. The photoperiod was also maintained on a schedule of 12 h light (08:00–20:00 h) and 12 h dark during the experiment. No mortality was recorded throughout the trial.

### Sample Collection and Analysis

At the termination of the feeding trial, fish were fasted for ~24 h to empty gut contents and anesthetized with overdose clove, *Syzygium aromaticum*, and oil (0.070 mg L^−1^). Subsequently, all the fish in each replicate were counted and weighed to calculate weight gain ratio (WGR) [100 × (final body weight (g) − initial body weight (g))/initial bodyweight (g)], specific growth rate (SGR) [100 × (Ln final weight (g) − Ln initial weight (g))/days], feed conversion ratio (FCR) [feed intake (g)/(final body weight (g) − initial body weight (g))], condition factor [final body weight (g)/fork length (cm^3^)], and survival rate (SR) [100 × final fish number/initial fish number] in each tank.

After the final weighing, nine fish from each dietary replicate tank were randomly selected to collect their blood samples from the caudal veins using a 5-ml syringe. The procured bloods from each replicate were pooled together and divided into two parts. The first part (1.5 mL) was left to clot at 4°C for 12 h into non-heparinized Eppendorf tubes and stored at −80°C after centrifuging at 3,500 × g and 4°C for 15 min using a refrigerated centrifuge (3−30KS, Sigma Laborzentrifugen GmbH, Osterode am Harz, Germany). The other part (1.5 mL) was transferred to the heparinized tube and stored at 4°C for blood cell investigations. After blood collection, the sampled fish were dissected on ice to obtain their liver and viscera in order to calculate the hepatosomatic index (HSI) [100 × hepatic weight (g)/body weight (g)] and viscerosomatic index (VSI) [100 × viscera weight (g)/body weight (g)], respectively.

Red blood cells (RBCs) and white blood cells (WBCs) were counted under a compound microscope at 100 × magnification using a Neubauer hemocytometer after blood dilution in Hayem and Turk solutions, respectively. The hematocrit (Hct) value was determined by placing the heparinized blood sample in glass capillary tubes and centrifuging for 3 min at 6,000 × g in a microhematocrit centrifuge (MC-150, Tomy Tech., USA) and measuring the packed cell volume (30). The hemoglobin concentration (Hb) was spectrophotometrically evaluated based on the cyanomethemoglobin method by measuring the absorbance at 540 nm (31). Mean cell volume (MCV) [10 × Hct (%)/RBCs (× 10^6^ cell mm^3^)], mean cell hemoglobin (MCH) [10 × Hb (g dL^−1^)/RBCs (× 10^6^ cell mm^3^)], and mean cell hemoglobin concentration (MCHC) [100 × Hb (g dL^−1^)/Hct (%)] were also calculated using the results determined from the RBC fraction (32). To determine differential leukocyte counts, blood smears from triplicate samples were first air-dried, fixed in 96% ethanol for 30 min, and examined for lymphocyte, neutrophil, eosinophil, monocyte, and basophil count under a compound microscope after staining by Giemsa solution for 30 min (33).

The serum activities of superoxide dismutase (SOD), glutathione-peroxidase (GPX), and catalase (CAT) were evaluated with a spectrophotometer (Cary 100 UV-vis, Agilent, Santa Clara, USA) using the commercial kits (Nanjing Jiancheng Bioengineering Institute, Nanjing, China) based on the manufacturer's instructions. One unit of SOD activity was specified as the quantity of enzyme required to induce half the maximum inhibition of the nitro blue tetrazolium reduction rate. One unit of GPX activity was defined as the amount of enzyme necessary to catalyze the oxidation of 1 μmol NADPH per min. One unit of CAT activity was defined as the quantity of enzyme needed to accelerate the decomposition of 1 mmol H_2_O_2_ per min. The serum concentration of malondialdehyde was also measured by thiobarbituric acid-reacting substance assay as described in Ohkawa et al. (34) by using acommercial kit (Nanjing Jiancheng Bioengineering Institute, Nanjing, China).

Serum lysozyme activity was measured according to the turbidimetric assay described by Ellis (35). Briefly, 25 μL of serum sample was added to 175 μL of *Micrococcus luteus* (Merck) suspension at a concentration of 0.2 mg mL^−1^ in 0.5 M phosphate buffer saline (pH 6.2). The reduction in optical density (OD) was recorded at 530 nm after 1 and 5 min at 22°C using a microplate reader (Labsystems Oy, Helsinki, Finland). A unit of lysozyme was defined as the amount of enzyme causing 1% decrease in absorbance during the incubation time of 4 min: [100 × (OD 1 min − OD 5 min)/OD 1 min] and expressed as unit mL^−1^ serum.

Immunoglobulin M (IgM) concentrations in the serum was measured by the sandwich enzyme-linked immunosorbent assay (ELISA) described by Lund et al. (36) with some modifications. Briefly, flat-bottomed 96-well plates were coated with serially diluted (1:200) serum samples and incubated overnight at 4°C. The wells were blocked with 5% skim milk for 2 h at room temperature after three-time washing with buffered phosphate-buffered saline Tween-20 (PBST; 50 mM sodium phosphate, pH 7.4, containing 150 mM NaCl and 0.1% Tween-20). The wells were then washed three times with PBST, filled with antiserum (100 μL at a 1:2,000 dilution), incubated for 1.5 h at 37°C, and rinsed again with PBST. The secondary anti-mouse antibody was also incubated in the identical conditions. An aliquot (100 μL) of 0.42 mM solution of O-phenylenediamine dihydrochloride in 100 mM citrate/sodium acetate buffer (pH 5.4) containing 0.03% hydrogen peroxide was added to each well. The reaction was stopped after 30 min dark incubation at room temperature by adding 25 μL of 2 M H_2_SO_4_ to each well. Negative controls were consisted as samples without a primary antibody. IgM activity was determined by subtracting the measured mean absorbance of the wells from the mean absorbance of the negative controls for each plate at 490 nm using an automated microplate reader (800 TS, Bio-Tech Instruments, Winooski, USA). Results were presented as mg dL^−1^.

The level of complement component C3 in serum was determined using a C3 kit (Pars Azmun Co. Ltd., Karaj, Iran). Methods for C3 activity analysis included measurement of the increase in turbidity after immunity response of C3 and its increased antibody (37). Results were presented as C3 mg dL^−1^. The level of complement components C4 in serum was determined using a C4 kit (Pars Azmun Co. Ltd., Karaj, Iran). Methods for C4 activity analysis was included measurement of the increase in turbidity after immunity response of C4 and its increased antibody (37). Results were presented as C4 mg dL^−1^.

### Statistical Analysis

Statistical analysis was performed using SPSS 26.0 package (SPSS Inc., Chicago, USA). All experiments and analyses were carried out in triplicate. Orthogonal polynomial contrasts were used to assay the linear and quadratic effects of FA-coated nanochitosan supplementation on rainbow trout. A *P* < 0.05 was considered as significant level. Results were expressed as mean ± SEM.

## Results

### Nanoparticle Properties

**Figure 1:**
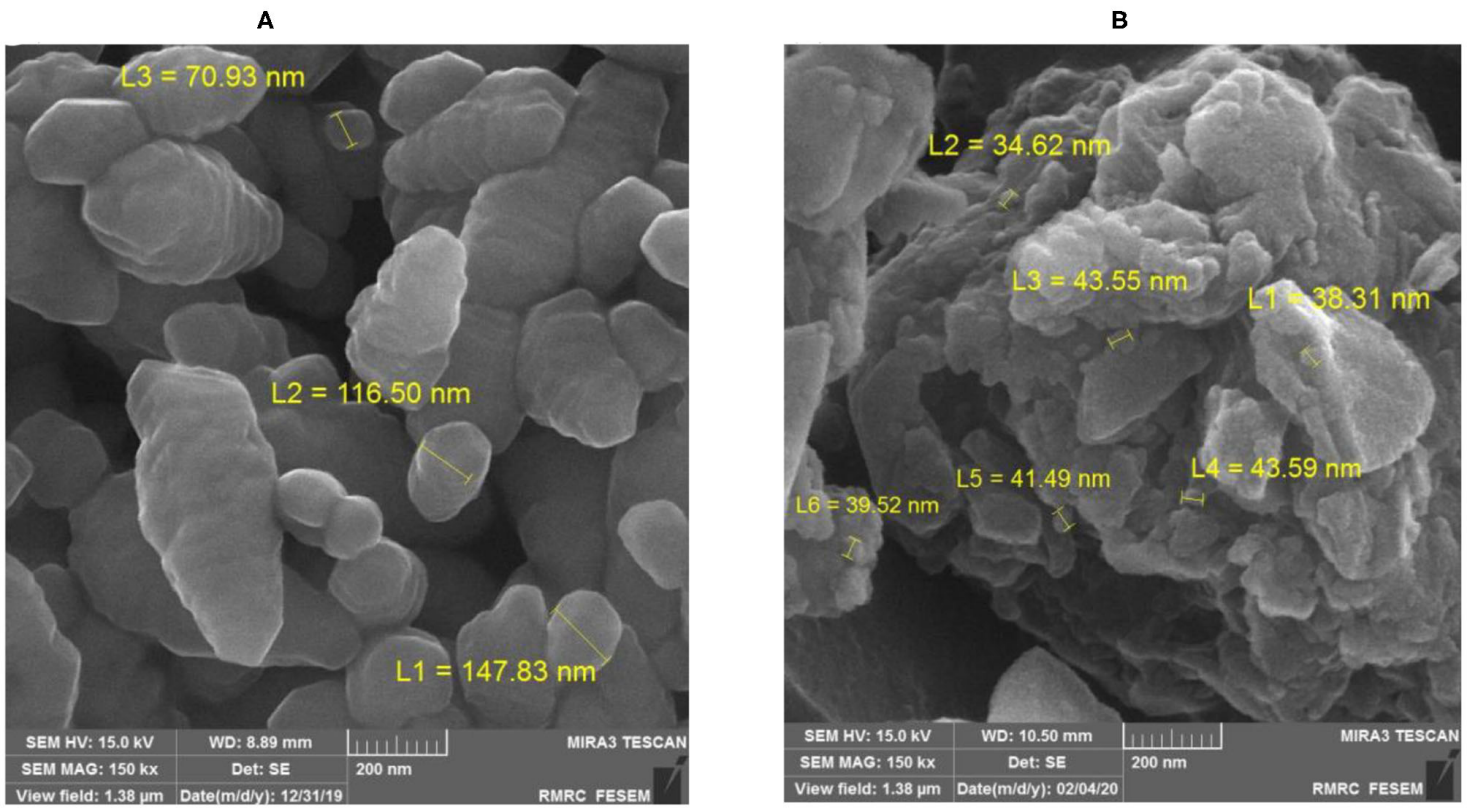
Scanning electron microscope image of chitosan nanoparticles before **(A)** and after **(B)** loading with folic acid (at 15 kV and 150 k × magnification).

**Figure 2:**
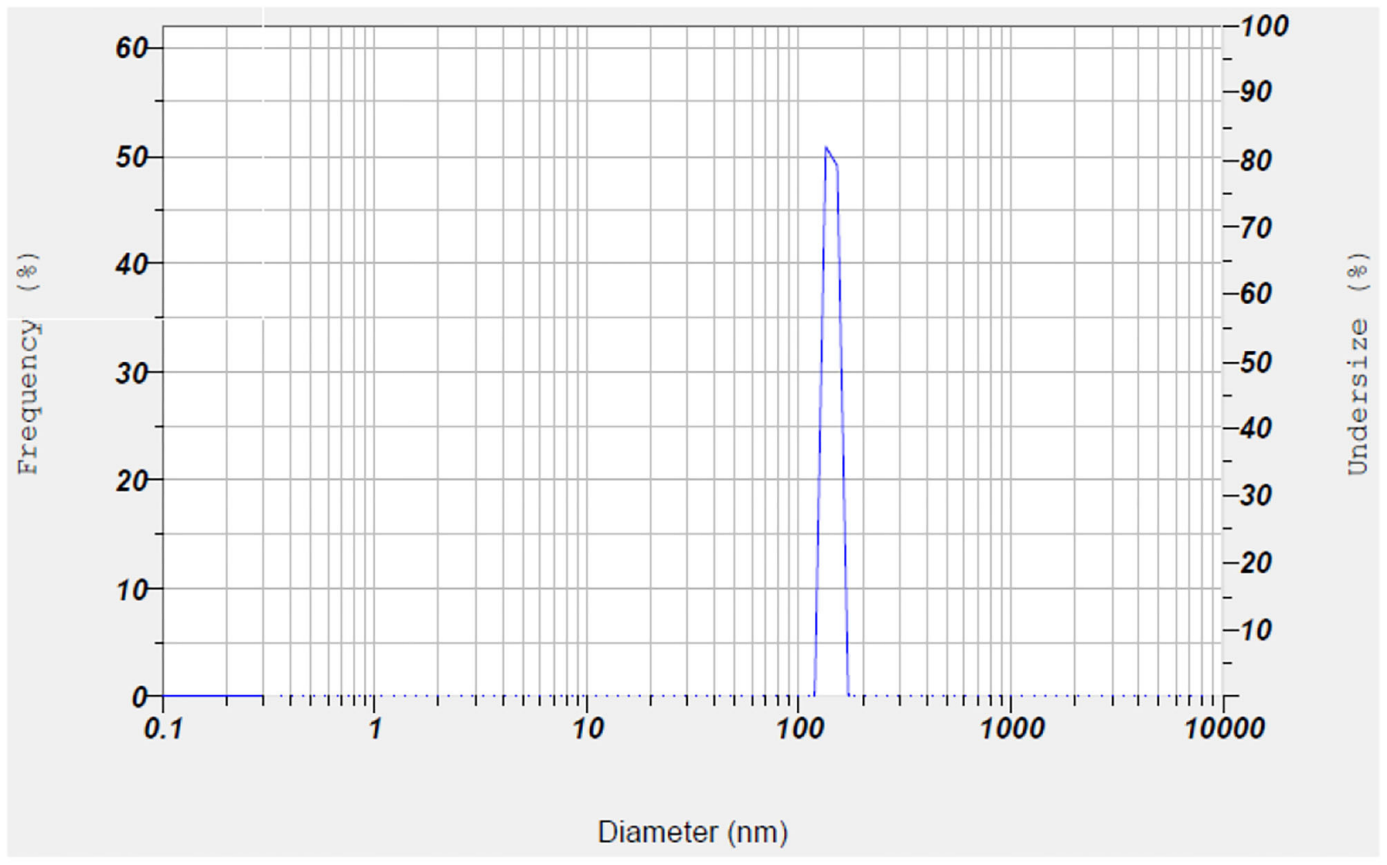
Particle size distribution of chitosan/folic acid nanoparticle.

The morphological properties of chitosan nanoparticles before (a) and after (b) loading with folic acid are given in Figure 1. Different-sized nanoparticles had continuous, spherical, and uniform structure and rough surface. The average size distribution of the chitosan nanoparticles coated with FA was found to be 134.30 ± 8.20 nm with major percentage of nanoparticles corresponding to the peak over 133.9 nm (Figure 2). Pure chitosan had the zeta potential value of 5.6 ± 0.1 mV, while the chitosan coated with folic acid had the zeta potential value of 6.5 ± 0.1 mV. The encapsulation efficiency and loading capacity of FA-coated nanoparticle were also 37.74 ± 0.91 and 7.29 ± 1.34%, respectively.

**Figure 3:**
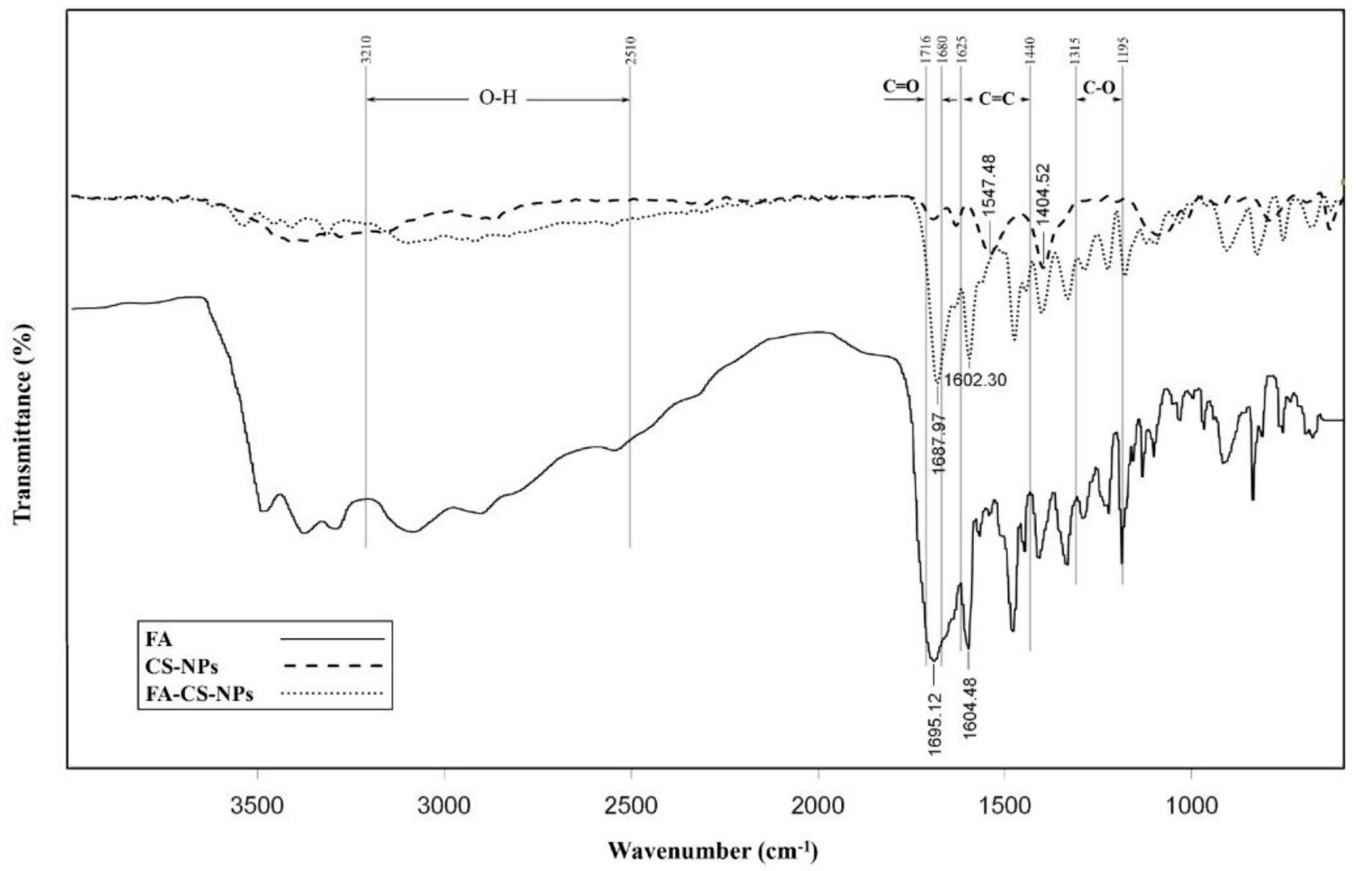
Fourier transform infrared spectra of folic acid (FA), chitosan nanoparticles (CS-NPs), and folic acid coated chitosan nanoparticles (FA-CS-NPs).

Figure 3 shows the FTIR spectra of FA, nanochitosan, and FA-coated nanochitosan. The range between two peaks of 1,546 ± 48 and 1,403 ± 52 cm^−1^ indicates strong bond vibrations of NH and NH_2_, respectively. The peak assignment in the FTIR spectrum illustrated N–H stretch at 3,505–3,208 cm^−1^; C–H stretch peak at 2,985–2,836 cm^−1^; and C–O stretch at 1,307–1,207 cm^−1^ in the chitosan nanoparticle. The O–H stretch at 3,210–2,510 cm^−1^; C=O stretch at 1,716–1,680 cm^−1^, C–O stretch at 1,315–1,195 cm^−1^, and C=C stretch in aromatic compounds at 1,625–1,440 cm^−1^ were also found in the FA molecule. The folic acid diagram has also severed declines at 1695.12 and 1604.48 cm^−1^ which represents the COOH and C–C bonds, respectively. In comparison with the nanochitosan diagram, the FA-coated nanochitosan spectra have a significant difference at 1687.97 cm^−1^, which may be due to the formation of the amide bond between the amino group of chitosan and the carboxyl group of folic acid. These results confirmed that folic acid was chemically conjugated to the chitosan backbone and could be used for its possible oral delivery.

### Fish Growth Performance

The growth performance and survival rate of fingerling rainbow trout fed on diets supplemented with different levels of folic acid as FA-coated nanochitosan is depicted in Table 2. During the 8-week bioassay, no mortality was recorded in experimental tanks and the SR was 100%. However, an increase in the level of dietary FA-coated nanochitosan linearly enhanced the WGR and SGR with the highest values in fish fed the 1.00-mg kg^−1^ FA-coated nanochitosan-supplemented diet (*P* < 0.05). Conversely, FCR linearly decreased and reached to the lowest value of 1.29 ± 0.08 in fish fed with 1.00 mg kg^−1^ FA-coated nanochitosan-supplemented diet (*P* < 0.05). Fish fed with the 0.25 FA-coated nanochitosan-supplemented diet showed the highest CF of 1.10 ± 0.05 g cm^−3^ (*P* < 0.05), while no difference was found between them and those fed the higher levels of FA-coated nanochitosan (*P* > 0.05). No significant difference was also found in HSI and VSI of fish among the experimental treatments.

**Table 2:** Growth performance and feed utilization of fingerling rainbow trout, *Oncorhynchus mykiss*, fed diets containing varying levels of folic acid-coated nanochitosan^*^.

	**Dietary FA-coated nanochitosan (mg kg**^**−1**^ **diet)**					**ANOVA**	**Linear trend**	**Quadratic trend**
	**0**	**0.10**	**0.25**	**0.50**	**1.00**			
SR	100.00	100.00	100.00	100.00	100.00	0.000	0.000	0.000
WGR (%)	161.25 ± 2.86	187.07 ± 13.35	203.72 ± 2.53	209.65 ± 9.66	223.34 ± 14.00	0.001	0.000	0.208
SGR (% d^−1^)	1.71 ± 0.02	1.88 ± 0.08	1.98 ± 0.01	2.02 ± 0.06	2.09 ± 0.08	0.001	0.000	0.134
FCR	1.64 ± 0.03	1.48 ± 0.01	1.35 ± 0.02	1.31 ± 0.06	1.29 ± 0.08	0.000	0.000	0.048
CF (g cm^−3^)	1.01 ± 0.01	1.02 ± 0.02	1.10 ± 0.05	1.04 ± 0.02	1.08 ± 0.04	0.046	0.044	0.352
HSI (%)	1.20 ± 0.30	1.19 ± 0.10	1.31 ± 0.24	1.19 ± 0.16	1.20 ± 0.15	0.969	0.977	0.705
VSI (%)	8.53 ± 1.06	9.03 ± 0.54	10.07 ± 1.59	8.85 ± 0.90	8.80 ± 0.99	0.664	0.891	0.265

* *Values are presented as means ± SEM (n = 9). SR, survival rate; WGR, weight gain ration; SGR, specific growth rate; FCR, feed conversion ratio; CF, condition factor; HIS, hepatosomatic index; VSI, viscerosomatic index*.

### Hematological Responses

The changes in hematological parameters of fingerling rainbow trout after feeding with different levels of FA-coated nanochitosan are presented in Table 3. There were a linearly and quadratically decreasing response in WBC counts as the level of dietary FA-coated nanochitosan increased and reached the lowest value in fish fed with the 0.25-mg FA-coated nanochitosan kg^−1^ diet (*P* < 0.05). In contrast, linearly increasing trends were observed in RBC counts, Hb concentration, and Hct of fish fed with increasing levels of dietary FA-coated nanochitosans. However, no significant difference was recorded in MCV, MCH, and MCHC of fish fed with different levels of FA-coated nanochitosan. There were also no significant differences in differential leukocyte cell counts including neutrophils, lymphocytes, monocytes, and eosinophils, among the experimental treatments.

**Table 3:** Hematological parameters of fingerling rainbow trout, *Oncorhynchus mykiss*, fed diets containing varying levels of folic acid-coated nanochitosan^*^.

	**Dietary FA-coated nanochitosan (mg kg^−1^ diet)**					**ANOVA**	**Linear trend**	**Quadratic trend**
	**0**	**0.10**	**0.25**	**0.50**	**1.00**			
WBC (×10^6^ mm^−3^)	4.20 ± 0.22	3.83 ± 0.12	3.80 ± 0.43	4.83 ± 0.34	5.10 ± 0.14	0.002	0.001	0.010
RBC (×10^6^ mm^−3^)	1566.67 ± 53.12	1740.00 ± 106.14	1686.67 ± 30.91	1698.33 ± 52.65	1723.33 ± 18.86	0.048	0.016	0.847
Hb (g dL^−1^)	7.83 ± 0.17	8.63 ± 0.40	8.40 ± 0.08	8.42 ± 0.15	8.62 ± 0.06	0.021	0.017	0.126
Hct (%)	41.00 ± 1.63	45.67 ± 3.09	45.33 ± 0.47	45.33 ± 1.25	46.67 ± 1.25	0.048	0.019	0.205
MCV (fL)	261.67 ± 1.70	261.85 ± 2.49	268.86 ± 6.80	267.00 ± 0.94	270.67 ± 5.44	0.052	0.701	0.304
MCH (pg)	50.03 ± 0.68	49.58 ± 0.69	49.81 ± 1.19	49.57 ± 1.81	50.01 ± 0.25	0.178	0.644	0.111
MCHC (g dL^−1^)	19.13 ± 0.39	18.90 ± 0.39	18.53 ± 0.39	18.57 ± 0.59	18.47 ± 0.39	0.051	0.111	0.594
Neutrophils (%)	14.33 ± 1.25	12.67 ± 0.94	13.67 ± 1.25	16.33 ± 1.25	15.00 ± 0.82	0.068	0.073	0.448
Lymphocytes (%)	81.00 ± 2.45	81.33 ± 1.70	81.33 ± 0.94	77.33 ± 2.05	77.67 ± 0.47	0.076	0.018	0.391
Monocytes (%)	4.00 ± 0.82	5.00 ± 0.82	4.67 ± 0.47	5.67 ± 0.47	5.67 ± 0.94	0.191	0.034	0.737
Eosinophils (%)	0.67 ± 0.47	1.00 ± 0.82	0.33 ± 0.47	0.67 ± 0.47	1.67 ± 0.47	0.239	0.211	0.145

* *Values are presented as means ± SEM (n = 9). WBC, white blood cell, RBC, red blood cell; Hb, hemoglobin; Hct, hematocrit; MCV, mean corpuscular volume; MCH, mean corpuscular hemoglobin; MCHC, mean corpuscular hemoglobin concentration*.

### Serum Antioxidant Responses

Table 4 illustrates the serum antioxidant status of fingerling rainbow trout fed the diet supplemented with different levels of FA-coated nanochitosan. After 8 weeks of feeding trial, the activities of CAT and SOD significantly linearly increased with increasing dietary level of FA-coated nanochitosan and reached the highest value in fish fed with 0.50 mg FA-coated nanochitosan (*P* < 0.05). GPX also illustrated a similar pattern with the maximum values of 193.67 ± 3.40 U L^−1^ in fish fed with 1.00 mg kg^−1^ FA-coated nanochitosan-supplemented diet (*P* < 0.05). In contrast, enhancing dietary FA-coated nanochitosan supplementation significantly declined the serum MDA content to the minimum of 74.00 ± 3.74 μmol L^−1^ in fish fed with 1.00 mg kg^−1^ FA-coated nanochitosan (*P* < 0.05).

**Table 4:** Antioxidant status of fingerling rainbow trout, *Oncorhynchus mykiss*, fed diets containing varying levels of folic acid coated nanochitosan^*^.

	**Dietary FA-coated nanochitosan (mg kg**^**−1**^ **diet)**					**ANOVA**	**Linear trend**	**Quadratic tread**
	**0**	**0.10**	**0.25**	**0.50**	**1.00**			
CAT (U L^−1^)	80.33 ± 3.86	100.00 ± 8.60	102.00 ± 6.48	112.00 ± 6.48	110.33 ± 4.64	0.003	0.000	0.062
SOD (U L^−1^)	39.33 ± 1.25	41.00 ± 2.94	44.00 ± 5.10	47.00 ± 0.82	44.00 ± 2.45	0.161	0.041	0.256
GPX (U L^−1^)	148.67 ± 11.90	147.00 ± 15.58	158.00 ± 9.93	167.00 ± 18.83	193.67 ± 3.40	0.029	0.004	0.144
MDA (μmol L^−1^)	96.33 ± 2.49	91.00 ± 2.45	85.00 ± 2.45	76.67 ± 6.02	74.00 ± 3.74	0.001	0.000	0.766

* *Values are presented as means ± SEM (n = 9). CAT, catalase; GPX, glutathione peroxidase; SOD, superoxide dismutase; MDA, malondialdehyde*.

### Serum Immunological Responses

As shown in Table 5, the LA of fingerling rainbow trout was linearly augmented with increasing dietary FA-coated nanochitosan to the highest value in fish fed the diet supplemented with 1.00 mg kg^−1^ FA-coated nanochitosan (*P* < 0.05). There were also linearly and quadratically increasing trends in IgM content as well as complement component C3 and C4 activities by increasing the supplementation of nanochitosan coated with FA and reached their peaks in fish fed the diet supplemented with 0.50 mg kg^−1^ FA-coated nanochitosan (*P* < 0.05).

**Table 5:** Immune response of fingerling rainbow trout, *Oncorhynchus mykiss*, fed diets containing varying levels of folic acid-coated nanochitosan^*^.

	**Dietary FA-coated nanochitosan (mg kg**^**−1**^ **diet)**					**ANOVA**	**Linear trend**	**Quadratic trend**
	**0**	**0.10**	**0.25**	**0.50**	**1.00**			
LA (U ml^−1^)	25.33 ± 1.25	26.00 ± 2.16	31.33 ± 1.70	33.67 ± 4.50	35.00 ± 2.45	0.013	0.001	0.817
IgM (mg dL^−1^)	45.67 ± 4.64	50.67 ± 5.44	63.33 ± 5.91	75.67 ± 2.49	54.67 ± 2.49	0.000	0.001	0.001
C3 (mg dL^−1^)	15.33 ± 2.62	19.67 ± 2.87	24.67 ± 2.62	30.67 ± 1.25	22.67 ± 1.25	0.001	0.006	0.001
C4 (mg dL^−1^)	42.33 ± 2.62	50.00 ± 4.32	56.00 ± 4.55	66.00 ± 2.94	48.33 ± 3.40	0.001	0.000	0.003

* *Values are presented as means ± SEM (n = 9). LA, lysozyme activity; IgM, immunoglobulin M; C3, complement component 3; C4, complement component 4*.

## Discussion

Folate is a key component with profound impact on the cellular metabolism, growth, and proliferation by providing one carbon unit for numerous biochemical reactions involved in the synthesis of DNA, amino acids, creatine, and phospholipids (38, 39). In the current research, the growth performance was poor in fish fed the FA-deficient diet and enhanced by increasing the dietary FA-coated nanochitosan, which clearly indicated that FA coated on the chitosan nanoparticles could supply the rainbow trout requirement for normal growth. Fish fed diet supplemented with 1.00 mg kg^−1^ FA-coated nanochitosan in the present study had also the lowest FCR, suggesting the complementary role of FA in feed utilization of rainbow trout. However, no mortality and deficiency signs were recorded in rainbow trout fed the basal diet in the current research which could be attributed to the synthesis of folic acid by intestinal bacteria (12). No FA deficiency signs were also reported in juvenile grouper (14) and hybrid tilapia (40).

Morphometric indices such as HSI, VSI, and CF are commonly used as important indicators for evaluating the nutritional and health status of fish (41). In the current study, no significant difference was recorded in HSI and VSI of rainbow trout with increment in dietary FA-coated nanochitosan concentrations. Consistent with present results, lower HSI was reported in Siberian sturgeon (16), blunt snout bream (15), grouper (14), and rainbow trout (42) fed the FA-supplemented diet. This inconsistency in the findings may be related to the lower physiological requirement of rainbow trout compared to the other species or higher bioavailability of vitamin B9 after coating on chitosan nanoparticles (physical form used in this research) which can meet its requirements under lower concentrations. A linear and significant increment was recorded in the CF of fish by increasing dietary FA-coated nanochitosan up to 0.25 mg kg^−1^ diet, which shows the appropriateness of this level of FA after coating on chitosan nanoparticles for rainbow trout.

The FA has come into focus in recent years due to its essential role in hematopoietic activities which are widely used as powerful diagnostic tools for monitoring the health status of farmed fish (43, 44). In the present study, RBC count and Hct of fish fed diet supplemented with different levels of FA-coated nanochitosan were significantly higher than those fed the basal diet, indicating the supplementary role of FA coated on nanochitosan for the normal deviation and maturation processes of erythrocytes in rainbow trout probably by participating in purine and pyrimidine biosynthesis (13, 45). FA also increases the mitochondrial synthesis of glycine from serine that is required for catalyzing the first step of hemoglobin production (46, 47). This may justify the higher Hb level of rainbow trout fed diet supplemented with FA-coated nanochitosan in the present study compared to those fed the basal diet, although glycine evaluation could provide more accurate information in this regard. No significant difference in MCV, MCH, and MCHC between rainbow trout-fed diets supplemented with different FA-coated nanochitosan and those fed the basal diet illustrated that the increase in hemoglobin concentration is associated with an increment in the erythrocyte count of rainbow trout and FA coated on chitosan nanoparticles has a similar effect on the synthesis of both factors.

It has been generally accepted that WBC count reveals the health status of fish in response to changes related to age, water quality, disease, and nutrition (44, 48). In spite of no significant difference in differential leukocyte counts among the experimental treatments, WBC count decreased as the dietary concentration of FA-coated nanochitosan increased up to 0.25 mg kg^−1^ diet, implying that dietary FA meets the physiological needs of rainbow trout in a way that reduced the necessity for higher leukocyte synthesis under cultural conditions. These results agreed with those reported by Abasian et al. (49) that dietary FA decreased RBCs of rainbow trout. However, increase in WBC count by increasing the dietary concentration of FA-coated nanochitosan from 0.25 to 1.00 mg kg^−1^ diet shows that high levels of dietary FA may affect the physiological balance of rainbow trout by increasing the necessity for white blood cell synthesis. More studies on this regard are required to confirm the precise mechanism between WBC and dietary FA supplementation.

To avoid the negative effects of naturally producing reactive oxygen species, fish like other aerobic animals developed multiple approaches to scavenge excess free radicals that initially begin with the activity of antioxidant enzymes like SOD, CAT, and GPX (50). Therefore, activities of these antioxidant enzymes could serve as a biomarker of the antioxidant defense status of the organism. SOD is one of the antioxidant proteins that catalyze the rapid dismutation of superoxide radicals (O_2−_) into molecular oxygen (O_2_) and hydrogen peroxide (H_2_O_2_), while GPX and CAT promote the conversion of H_2_O_2_ to water and oxygen, thereby providing cellular defense against oxidative damages (51–53). In the present study, SOD and CAT activities increased with increasing dietary FA-coated nanochitosan supplementation up to 0.50 mg kg^−1^ and thereafter tended to decline, which is in accordance with studies on murrel, *Channa punctata* (10), grouper (14), and Siberian sturgeon (16). Furthermore, GPX activity was amplified by supplementing dietary FA-coated nanochitosan and reached to the highest level of 193.67 ± 3.40 U L^−1^ in fish fed with 1.00 mg kg^−1^ FA-coated nanochitosan-supplemented diet, suggesting that folic acid after coating on chitosan nanoparticle could improve the antioxidant capacity of rainbow trout by enhancing the activity of antioxidant enzymes. On the other hand, lower SOD and CAT activities in the serum of rainbow trout fed the 1.00-mg kg^−1^ FA-coated nanochitosan diet suggest that deficient or excess supplementation of dietary FA-coated nanochitosan can reduce the antioxidant capacity of rainbow trout.

Excessive levels of reactive oxygen species-induced oxidative damage could be also evaluated by the content of MDA, the most abundant product of lipid peroxidation that may alter the activity of many proteins by inducing the cross-linkages between the polypeptides (50, 54). In agreement with previous studies (5, 10), decreased levels of MDA in the present research by increasing dietary FA-coated nanochitosan illustrated that the process of lipid peroxidation could be reduced by supplementing the FA after coating on chitosan nanoparticles, probably due to its boosting effect on the activity of antioxidant enzymes. Although the underlying mechanisms by which dietary FA declines lipid peroxidation of freshwater fish remained unclear, Shi et al. (55) expressed that folic acid could enhance the antioxidant capacity partly by upregulating the nuclear factor erythroid 2-related factor 2 (Nrf2) gene expression in the intestine which acts as an emerging regulator of cellular resistance to oxidants in the liver and intestine. However, understanding the precise mechanism in fish requires further investigations.

Innate immunity is an important component of the host defense that initiates within hours to provide a quick line of defense against infection (56). Among innate immune components, LZ activity is one of the earliest known antibacterial proteins that can improve the bacterial phagocytosis by splitting the peptidoglycan layer in their cell walls (57). Shi et al. (55) stated that FA deficiency has negative effects on the innate immunity capacity of grass carp by attenuating the LZ activity in the fish intestine. It was also reported that dietary FA supplementation could improve the LZ activity of Siberian sturgeon (16) and grouper (14). Moreover, chitosan is used as an immunostimulant against bacterial disease of salmonid fish by inducing the nonspecific mechanisms of defense (58). In this study, supplementing chitosan nanoparticles coated with FA increased the serum LZ activity, implying that FA and chitosan in the nanoscale supplementation could enhance the innate immune aptitude of rainbow trout probably by the ability of FA to carry one carbon group involved in the metabolism of certain amino acids necessary for LZ activity (43) or amino moieties of chitosan required to induce the innate immunity system (28).

The complement system is another part of the immune system composed of many proinflammatory proteins that play pivotal roles in the innate defense against common pathogens (59, 60). Among these proteins, C3 is critical for activation of the complement system as a whole, while C4 is the major protein of the classical cascade (61). As shown earlier, FA deficiency declined the C3 content in the intestine and gill of grass carp (9, 55). It was also reported that dietary FA improves both the serum C3 and C4 contents of blunt snout bream (15). In addition, Cha et al. (58) expressed that chitosan could be applied as an immunostimulant for salmonids against bacterial diseases due to its amino moieties that induce the nonspecific defense mechanisms. In the present study, the serum C3 and C4 of rainbow trout were significantly increased by dietary FA-coated nanochitosan supplementation and reached the highest response in fish fed with 0.50 mg kg^−1^ FA-coated nanochitosan-supplemented diet, suggesting that chitosan and FA may have synergetic effects on the innate immune system of rainbow trout. However, further studies are needed to figure out the detailed process by which dietary FA and chitosan nanoparticles improve these responses.

IgM is the preponderant immunoglobulin molecule in fish secreted by B lymphocytes that leads to the opsonization of pathogens and simplification of their phagocytosis (62). Fish fed diet supplemented with dietary FA-coated nanochitosan in the present study had higher IgM contents in their serum compared to those fed the basal diet which enhanced the serum IgM content, implying that FA and chitosan nanoparticles stimulate the IgM synthesis in rainbow trout, probably by increasing the B lymphocyte proliferation. It is specified that chitosan-supplemented diet significantly increases the IgM content in the serum of loaches (63). FA supplementation also increased the IgM content of Siberian sturgeon after an 8-week feeding trial (16). These findings all show that FA and chitosan can strengthen the immune system by increasing IgM levels, although further investigation is needed to understand the specific pathway by which dietary FA and nanochitosan amplify these responses.

In conclusion, findings of the current study corroborated the positive effect of dietary FA-coated nanochitosan on growth performance and feed utilization of rainbow trout with the best responses in fish fed the highest dietary FA-coated nanochitosan. An increasing trend of WBC count, Hb concentration, and hematocrit was also observed in rainbow trout fed diet supplemented with higher dietary FA-coated nanochitosan. Antioxidant enzymes including CAT and SOD also reached their highest activities in fish fed diet supplemented with 0.50 mg kg^−1^ FA-coated nanochitosan, while the highest activity of GPX was found in fish fed diet supplemented with the 1.00-mg kg^−1^ FA-coated nanochitosan. LA was amplified by increasing FA-coated nanochitosan, while the highest IgM, C3, and C4 contents were found in rainbow trout fed diet supplemented with 0.50 mg kg^−1^ FA-coated nanochitosan. Therefore, it can be concluded that FA coated on chitosan nanoparticles improves the performance, antioxidant properties, and immune responses of rainbow trout.

## Data Availability Statement

The raw data supporting the conclusions of this article will be made available by the authors, without undue reservation.

## Ethics Statement

The procedure of the present study met the ethical legal principles and professional requirements of the ethics committee for conducting medical research in Iran (approval ID: IR.IAU.SRB.REC.1399.107).

## Author Contributions

SF: field study. HR: supervision. SM: data analysis. MS: analysis advisement. All authors contributed to the article and approved the submitted version.

## Conflict of Interest

The authors declare that the research was conducted in the absence of any commercial or financial relationships that could be construed as a potential conflict of interest.
